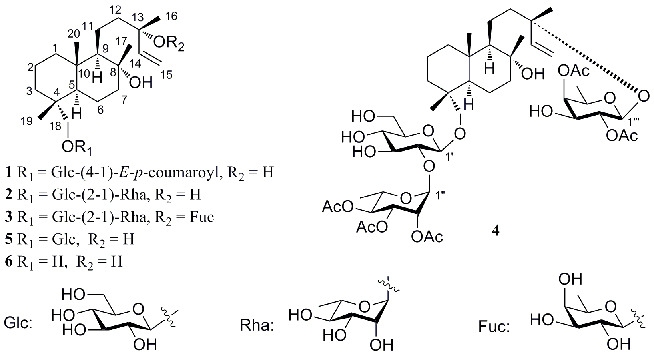# Four new labdane-type diterpenoid glycosides from *Diplopterygium laevissimum*

**DOI:** 10.1007/s13659-012-0022-3

**Published:** 2013-03-02

**Authors:** Ming-Ming Li, Kou Wang, Juan He, Li-Yan Peng, Xuan-Qin Chen, Xiao Cheng, Qin-Shi Zhao

**Affiliations:** State Key Laboratory of Phytochemistry and Plant Resources in West China, Kunming Institute of Botany, Chinese Academy of Sciences, Kunming, 650201 China

**Keywords:** *Diplopterygium laevissimum*, labdane-type diterpenoid glycosides, laevissiosides

## Abstract

Four new labdane-type diterpenoid glycosides, laevissiosides A-D (**1**–**4**) were isolated from the 95% ethanol extract of *Diplopterygium laevissimum* (Christ) Nakai, along with two known analogues, 18- *β*-D-glucopyranosyl ester-sclareol (**5**) and 18-hydroxy-sclareol (**6**). The structures of compounds **1**–**4** were elucidated by extensive 1D and 2D NMR spectroscopy as well as high-resolution MS analyses. All isolated compounds were evaluated for their cytotoxic effects.

## Introduction

*Diplopterygium laevissimum* (Christ) Nakai, belonging to the Gleicheniaceae family, is widely distributed in south China. Its rhizome has been used for treating hemostasis, stomach, and epistaxis as Chinese herbal medicine.[1] Many clerodane- and labdane-type diterpenoid glycosides, which are commonly glycosidated at C-13 in ferns, have been isolated from this family.[2–8] Previous research showed that some clerodane-type diterpenoid glycosides isolated from *Dicranopteris* species could accelerate the growth of the stems of lettuce and inhibit the root growth.[5]

Our previous chemical studies have led to the isolation of two highly oxygenated phenolic derivatives and some clerodane-type diterpenoid glycosides from *Dicranopteris* and an *ent*-kaurene diterpenoid glycoside from *Hicriopteris*.[6,9–11] As a systematic research work on the bioactive constituents from the ferns, the whole plant of *D. laevissimum* had been studied, which led to the isolation of four new labdane-type diterpenoid glycosides (**1**–**4**), along with two known analogues (**5** and **6**). All of these showed no *in vitro* cytotoxicity against five human cancer cell lines (HL-60, SMMC-7712, A-549, SK-BR-3 and PANC-1). Herein, the isolation and structure elucidation of compounds **1**–**4** were described.

## Results and Discussion

Compound **1** was obtained as a white amorphous powder. The molecular formula $C_{35}H_{52}O_{10}$ was established by the HRESIMS (631.3471 $[M - H]^-$; calcd. 631.3482),

**4**

**1** $R_1$ = Glc-(4-1)-*E*-*p*-coumaroyl, $R_2$ = H
**2** $R_1$ = Glc-(2-1)-Rha, $R_2$ = H
**3** $R_1$ = Glc-(2-1)-Rha, $R_2$ = Fuc
**5** $R_1$ = Glc, $R_2$ = H
**6** $R_1$ = H, $R_2$ = H

corresponding to ten degrees of unsaturation. The IR spectrum showed the presence of hydroxyl (3428 $cm^{-1}$) and carbonyl (1704 $cm^{-1}$) groups. The $^1H$ and $^{13}C$ NMR (Tables 1 and 2) indicated the existence of a set of signals for a hexose [anomeric signals at $\delta_H$ 4.30 (d, $J$ = 8.0 Hz); $\delta_C$ 104.6] and other 20 carbon resonances, including two olefinic carbons ($\delta_C$ 147.5 and 110.8) and three oxygen-bearing carbons ($\delta_C$ 73.4, 73.9 and 79.2). These data were very similar to those of 18-*β*-D-glucopyranosyl ester-sclareol (**5**), a known compound also isolated from this plant. However, detailed comparison the MS and NMR data of **1** with those of **5** revealed that **1** had one more *p*-coumaroyl group, which was attached to C-4′ of the sugar moiety as concluded from the HMBC (Figure 1) correlations of H-4′ ($\delta_H$ 4.84) with C-1″ ($\delta_C$ 167.4). The double bond of the *p*-coumaroyl group was suggested as *trans*- due to the coupling constant ($J$ = 15.6 Hz). Acidic hydrolysis of **1** gave D-glucose as sugar residue. The coupling constants of the anomeric proton ($J$ = 8.0 Hz) indicated the *β* configuration of glucosyl moiety. Assignment of glycosidic protons system was achieved by analysis of $^1H$-$^1H$ COSY and HSQC. The location

*To whom correspondence should be addressed. E-mail: qinshizhao@mail.kib.ac.cn

**Table 1.** $^1$**H NMR data of compounds 1–4 (*δ* in ppm, *J* in Hz)**

| no. | **1**[a] | no. | **2**[a] | **3**[b] | **4**[a,c] |
|---|---|---|---|---|---|
| 1 | 1.66 (m); 0.95 (m) | 1 | 1.59 (m); 1.09 (m) | 1.59 (m); 1.04 (m) | 1.79 (m); 1.13 (m) |
| 2 | 1.63 (m); 1.42 (m) | 2 | 1.59 (m); 1.41 (m) | 1.60 (m); 1.45 (m) | 1.69 (m); 1.51 (m) |
| 3 | 1.56 (m); 1.27 (m) | 3 | 1.59 (m); 1.18 (m) | 1.50 (m); 1.30 (m) | 1.48 (m); 1.40 (m) |
| 5 | 1.48 (m) | 5 | 1.36 (m) | 1.38 (m) | 1.32 (m) |
| 6 | 1.55 (m); 1.25 (m) | 6 | 1.52 (m); 1.27 (m) | 1.55 (m); 1.36 (m) | 1.62 (m); 1.32 (m) |
| 7 | 1.70 (m); 1.26 (m) | 7 | 1.74 (m); 1.52 (m) | 1.75 (m); 1.47 (m) | 1.80 (m); 1.50 (m) |
| 9 | 1.16 (m) | 9 | 1.35 (m) | 1.24 (m) | 1.00 (m) |
| 11 | 1.33 (m) | 11 | 1.47 (m); 1.29 (m) | 1.50 (m); 1.36 (m) | 1.43 (m) |
| 12 | 1.73 (m); 1.55 (m) | 12 | 1.78 (m); 1.56 (m) | 1.62 (m); 1.51 (m) | 1.76 (m); 1.54 (m) |
| 14 | 5.91 (dd, 10.8, 17.6) | 14 | 5.89 (dd,10.5, 17.0) | 5.91 (dd, 10.8, 17.6) | 5.88 (dd, 10.8, 18.0) |
| 15 | 4.92 (d, 10.8); 5.19 (d, 17.6) | 15 | 5.15 (d, 17.0); 4.91(d, 10.5) | 5.17 (d, 17.6); 5.15 (d, 10.8) | 5.18 (m); 5.16 (m) |
| 16 | 1.20 (s) | 16 | 1.05 (s) | 1.09 (s) | 1.09 (s) |
| 17 | 1.10 (s) | 17 | 1.19 (s) | 1.32 (s) | 1.32 (s) |
| 18 | 3.45 (m); 3.25 (m) | 18 | 3.38 (m); 3.20 (m) | 3.48 (m); 3.21 (m) | 3.44 (m); 3.35 (m) |
| 19 | 0.75 (s) | 19 | 0.70 (s) | 0.76 (s) | 0.81 (s) |
| 20 | 0.82 (s) | 20 | 0.79 (s) | 0.82 (s) | 0.85 (s) |
| 1′ | 4.30 (d, 8.0) | 1′ | 4.27 (d, 7.5) | 4.25 (d, 7.2) | 4.42 (d, 7.2) |
| 2′ | 3.33 (m) | 2′ | 3.24 (m) | 3.45 (m) | 3.53 (m) |
| 3′ | 3.68 (m) | 3′ | 3.52 (m) | 3.46 (m) | 3.68 (m) |
| 4′ | 4.84 (t, 9.6) | 4′ | 3.26 (m) | 3.24 (m) | 3.36 (m) |
| 5′ | 3.48 (m) | 5′ | 3.48 (m) | 3.19 (m) | 3.35 (m) |
| 6′ | 3.54 (m); 3.58 (m) | 6′ | 3.79 (m); 3.62 (m) | 3.64 (m); 3.83 (m) | 3.68 (m); 3.84 (m) |
| 2″ | 6.36 (d, 15.6) | 1″ | 5.51 (d, 1.5) | 5.42 (d, 1.5) | 5.62 (d, 0.8) |
| 3″ | 7.64 (d, 15.6) | 2″ | 3.87 (m) | 3.91 (m) | 5.32 (m) |
| 5″/9″ | 7.55 (d, 8.8) | 3″ | 3.77 (m) | 3.74 (m) | 5.15 (m) |
| 6″/8″ | 6.90 (d, 8.8) | 4″ | 3.43 (m) | 3.40 (m) | 5.12 (m) |
| | | 5″ | 4.08 (m) | 4.06 (m) | 4.46 (m) |
| | | 6″ | 1.25 (d, 6.5) | 1.27 (d, 6.0) | 1.18 (d, 6.4) |
| | | 1‴ | | 4.28 (d, 8.0) | 4.52 (d, 8.0) |
| | | 2‴ | | 3.41 (m) | 5.37 (m) |
| | | 3‴ | | 3.40 (m) | 3.87 (m) |
| | | 4‴ | | 3.55 (m) | 4.92 (m) |
| | | 5‴ | | 3.52 (m) | 3.82 (m) |
| | | 6‴ | | 1.20 (d, 6.4) | 1.08 (d, 6.4) |

[a]Measured in acetone-$d_6$. [b]Determined in $CD_3OD$. [c]Acetyl groups $\delta_H$: (1.98, 2.06, 2.13, 2.13, 2.16).

of the sugar unit was established by the HMBC correlations of H-1′ ($\delta_H$ 4.30) with C-18 ($\delta_C$ 79.2).

The relative configuration of the aglycone was established on a ROESY experiment. The ROESY correlations (Figure 2) between H-5 and H-9 confirmed that these hydrogen atoms were *α*-oriented, while correlations of H-11/Me-17, H-11/Me-20, Me-17/Me-19, Me-17/Me-20, and Me-19/Me-20 indicated they were *β*-orientation. The absolute configuration of C-13 was inferred as *S* according to the chmical shift of C-13 ($\delta_C$ 73.4).[12–14] Therefore, the structure of **1** was determined as shown, named laevissioside A.

Compound **2**, a white amorphous powder, and its molecular formula, $C_{32}H_{56}O_{12}$, was determined on the basis of the HRESIMS (667.3454 $[M + Cl]^-$; calcd. 667.3460). The $^1$H and $^{13}$C NMR spectroscopic data of **2** were very similar to those of **5**, except for one more sugar moiety signals ($\delta_C$ 100.8, 72.0, 71.7, 73.7, 69.2, 18.3) presented in **2** which was further confirmed by mass spectra. Acidic hydrolysis of **2** gave D-glucose and L-rhamnose as sugar residues. The coupling

**Figure 1.** Selected HMBC correlations of compound **1** and **2**

constants ($\delta_H$ 4.27, $J$ = 7.5 Hz and $\delta_H$ 5.51, $J$ = 1.5 Hz) of anomeric protons of the two sugar moieties indicated the *β* configuration glucose and *α* configuration of rhamnose. The HMBC correlations between H-1″ ($\delta_H$ 5.51) and C-2′ ($\delta_C$ 77.5) identified a rhamnosyl (1→2) glucopyranosyl linkage. Furthermore, the sugar chain was linked to C-18 of the aglycone as inferred from the HMBC (Figure 1) correlation of

**Table 2.** $^{13}$**C NMR data of compounds 1-4 ( $\delta$ in ppm)**

| no. | 1[a] | no. | 2[a] | 3[b] | 4[a,c] |
|---|---|---|---|---|---|
| 1 | 39.8 $CH_2$ | 1 | 39.7 $CH_2$ | 40.4 $CH_2$ | 39.5 $CH_2$ |
| 2 | 18.5 $CH_2$ | 2 | 18.4 $CH_2$ | 18.8 $CH_2$ | 18.4 $CH_2$ |
| 3 | 36.6 $CH_2$ | 3 | 36.5 $CH_2$ | 37.0 $CH_2$ | 36.4 $CH_2$ |
| 4 | 37.9 C | 4 | 37.8 C | 38.3 C | 38.2 C |
| 5 | 49.6 CH | 5 | 48.9 CH | 50.7 CH | 51.2 CH |
| 6 | 21.0 $CH_2$ | 6 | 20.9 $CH_2$ | 21.4 $CH_2$ | 21.5 $CH_2$ |
| 7 | 44.6 $CH_2$ | 7 | 44.7 $CH_2$ | 44.8 $CH_2$ | 45.4 $CH_2$ |
| 8 | 73.9 C | 8 | 74.6 C | 75.4 C | 73.8 C |
| 9 | 62.1 CH | 9 | 60.9 CH | 62.0 CH | 62.9 CH |
| 10 | 40.0 C | 10 | 40.0 C | 40.4 C | 40.0 C |
| 11 | 20.1 $CH_2$ | 11 | 20.4 $CH_2$ | 20.5 $CH_2$ | 19.9 $CH_2$ |
| 12 | 46.5 $CH_2$ | 12 | 45.7 $CH_2$ | 44.8 $CH_2$ | 46.7 $CH_2$ |
| 13 | 73.4 C | 13 | 73.7 C | 81.8 C | 80.7 C |
| 14 | 147.5 CH | 14 | 147.0 CH | 145.0 CH | 143.5 CH |
| 15 | 110.8 $CH_2$ | 15 | 111.1 $CH_2$ | 115.2 $CH_2$ | 115.5 $CH_2$ |
| 16 | 28.1 $CH_3$ | 16 | 23.6 $CH_3$ | 23.0 $CH_3$ | 23.8 $CH_3$ |
| 17 | 24.6 $CH_3$ | 17 | 27.3 $CH_3$ | 23.7 $CH_3$ | 24.6 $CH_3$ |
| 18 | 79.2 $CH_2$ | 18 | 78.2 $CH_2$ | 79.4 $CH_2$ | 79.0 $CH_2$ |
| 19 | 17.9 $CH_3$ | 19 | 18.2 $CH_3$ | 18.2 $CH_3$ | 17.9 $CH_3$ |
| 20 | 16.2 $CH_3$ | 20 | 16.2 $CH_3$ | 16.5 $CH_3$ | 16.3 $CH_3$ |
| 1′ | 104.6 CH | 1′ | 102.9 CH | 103.5 CH | 102.5 CH |
| 2′ | 75.2 CH | 2′ | 77.5 CH | 78.0 CH | 75.9 CH |
| 3′ | 75.5 CH | 3′ | 79.6 CH | 79.8 CH | 79.3 CH |
| 4′ | 72.3 CH | 4′ | 71.9 CH | 72.0 CH | 72.1 CH |
| 5′ | 75.8 CH | 5′ | 76.8 CH | 77.9 CH | 77.5 CH |
| 6′ | 62.4 $CH_2$ | 6′ | 62.6 $CH_2$ | 62.7 $CH_2$ | 62.5 $CH_2$ |
| 1″ | 167.4 C | 1″ | 100.8 CH | 101.5 CH | 97.8 CH |
| 2″ | 115.2 CH | 2″ | 72.0 CH | 72.3 CH | 70.5 CH |
| 3″ | 146.1 CH | 3″ | 71.7 CH | 72.1 CH | 71.0 CH |
| 4″ | 121.8 C | 4″ | 73.7 CH | 74.0 CH | 74.0 CH |
| 5″/9″ | 116.7 CH | 5″ | 69.2 CH | 70.0 CH | 67.1 CH |
| 6″/8″ | 131.0 CH | 6″ | 18.3 $CH_3$ | 18.8 $CH_3$ | 17.6 $CH_3$ |
| 7″ | 160.8 C | 1‴ | | 99.6 CH | 97.1 CH |
| | | 2‴ | | 72.4 CH | 70.1 CH |
| | | 3‴ | | 75.2 CH | 71.1 CH |
| | | 4‴ | | 73.0 CH | 73.4 CH |
| | | 5‴ | | 71.5 CH | 69.5 CH |
| | | 6‴ | | 17.0 $CH_3$ | 16.9 $CH_3$ |

[a]Measured in acetone-$d_6$. [b]Determined in $CD_3OD$. [c]Acetyl groups ($\delta_C$: C=O 170.0, 170.4, 170.6, 171.0, 171.5; Me: 20.7, 20.8, 21.2, 21.2, 21.2).

H-1′ ($\delta_H$ 4.27) with C-18 ($\delta_C$ 78.2). Thus, the structure of **2** was elucidated and named as laevissioside B.

Compound **3**, obtained as a white amorphous powder, had the molecular formula $C_{38}H_{66}O_{16}$ as determined by the analysis of $^1$H, $^{13}$C, and DEPT NMR data and verified by the HRESIMS (813.4042 [M + Cl]$^-$; calcd. 813.4039). Compared with **2**, compound **3** had one more sugar moiety ($\delta_C$ 99.6, 72.4, 75.2, 73.0, 71.5, and 17.0) which was attached to C-13 as inferred from the HMBC correlations from H-1‴ to C-13. Acidic hydrolysis of **3** gave D-glucose, L-rhamnose, and D-fucose as sugar residues. They were in $\beta$, $\alpha$ and $\beta$ configurations, respectively, by the coupling constants ($\delta_H$ 4.25, $J$ = 7.2 Hz, $\delta_H$ 5.42, $J$ = 1.5 Hz, and $\delta_H$ 4.28, $J$ = 8.0 Hz) of their anomeric protons. The sugar moiety linked to C-13 was supposed to be fucose by the HMBC correlations of the anomeric proton H-1‴ ($\delta_H$ 4.28) with C-13 ($\delta_C$ 81.8). The linkage and location of the other two sugar moieties were suggested the same as compound **2** deduced from the HMBC correlations. Accordingly, the structure of **3** was established as shown, named laevissioside C.

The molecular formula of compound **4** was deduced as $C_{48}H_{76}O_{21}$ by the HRESIMS (1023.4557 [M + Cl]$^-$; calcd 1023.4567). The $^1$H and $^{13}$C NMR features of **4** were closely related to those of **3**. The only difference was that there were five more acetyl groups in **4**. The locations of acetyl groups were confirmed by the HMBC experiments. The correlations of H-2‴ ($\delta_H$ 5.37) with $\delta_C$ 170.4, H-4‴ ($\delta_H$ 4.92) with $\delta_C$ 170.0, H-2″ ($\delta_H$ 5.32) with $\delta_C$ 171.5, H-3″ ($\delta_H$ 5.15) with $\delta_C$171.0, and H-4″ ($\delta_H$ 5.12) with $\delta_C$ 170.6 indicated the five acetyl groups attached to C-2‴, C-4‴, C-2″, C-3″, and C-4″, respectively. Therefore, the structure of **4** was identified and named as laevissioside D.

**Figure 2.** Significant ROESY correlations of compounds **1**–**4**

Since the known compounds 18-$\beta$-D-glucopyranosyl ester-sclareol (**5**, $[\alpha]_D^{24.3}$ – 27.7) and 18-hydroxy-sclareol (**6**, $[\alpha]_D^{24.3}$ – 4.7), whose physical properites were quite difference with that reported,[15,16] were also isolated from this plant, compounds **1**–**4** should be labdane-type diterpenoid glycosides from the biogenic view.

All compounds isolated were evaluated for their cytotoxic activity against five human cancer cell lines, HL-60 myeloid leukemia, SMMC-7721 hepatocellular carcinoma, A-549 lung cancer, SK-BR-3 breast cancer, PANC-1 pancreatic cancer, applying the MTT method. However, all of the compounds were inactive, and they showed $IC_{50}$ values > 40 $\mu$M.

## Experimental Section

**General Experimental Procedures.** Optical rotations were measured on a Horiba SEPA-300 polarimeter. IR spectra were obtained by Tensor 27 FT-IR spectrometer with KBr pellets. The $^1$H and $^{13}$C NMR spectra were recorded on Bruker AV-400 spectrometers in acetone-$d_6$ at room temperature ($\delta$ in ppm, $J$ in Hz). FABMS was carried out on a VG Autospec-3000 spectrometer. HRESIMS was recorded with an API QSTAR Pulsar i spectrometer. Silica gel (200–300 mesh), Silica gel H (Qingdao Marine Chemical Ltd., China), and LiChroprep RP-18 silica gel (40–63 $\mu$m, Merck, Dramstadt, Germany) were used for column chromatography. Fractions were monitored by TLC and spots visualized by heating silica gel plates immersed with 15% $H_2SO_4$ in ethanol. Solvents were distilled prior to use. Preparative HPLC was performed on a Shimadzu

LC-8A preparative liquid chromatograph with Shimadzu PRC-ODS (K) column. Sephadex LH-20 (Amersham Pharmacia biotech, Sweden).

**Plant Material.** The aerial parts of *D. laevissimum* were collected from Pingbian, Yunnan Province, China in July 2007 and identified by Professor Xiao Cheng at Kunming Institute of Botany, Chinese Academy of Sciences. A voucher specimen (No. 200707A03) has been deposited in the State Key Laboratory of Phytochemistry and Plant Resources in West China, Kunming Institute of Botany, Chinese Academy of Sciences.

**Extraction and Isolation.** The dried and powdered plant materials (2.6 kg) were extracted with 95% ethanol (15.0 L, each 2 d) for three times. After evaporation of the solvent in vacuo, the concentrate was suspended into $H_2O$ and partitioned successively with ethyl acetate. The ethyl acetate extract (120 g) was chromatographed on a silica gel column eluted with $CHCl_3$-MeOH (1:0 to 5:5) to give five fractions 1–5. Fraction 2 was subjected to column chromatograph (CC) over silica gel (petroleum ether-acetone 8.5:1.5) and further purified by recrystallization to obtain **6** (20 mg). Fraction 3 was eluted with $CHCl_3$-MeOH (9:1) over silica gel CC then further purified by RP-18 and Sephadex LH-20 to yield **1** (3 g), **4** (500 mg), and **5** (2 g). Fraction 4 was subjected to ($CHCl_3$:MeOH = 8.5:1.5) and further purified by RP-18 and Sephadex LH-20 to afford **2** (8 mg) and **3** (20 mg).

**Laevissioside A:** amorphous powder; $[\alpha]_D^{19.2}$ – 50.4 (*c* = 0.14, MeOH). UV (MeOH) $\lambda_{max}$ (log $\varepsilon$): 314 (4.18), 227 (3.94), 211 (3.95), 200 (3.99), 192 (3.91) nm. IR (KBr): 3428, 2931, 1704, 1630, 1604, 1515, 1450, 1387, 1162, 1066, 1031 $cm^{-1}$. $^1H$ and $^{13}C$ NMR: see Table 1. FABMS (neg.) *m/z*: 631 $[M - H]^-$. HRESIMS (neg.) *m/z*: 631.3471 ($C_{35}H_{51}O_{10}$; calcd. 631.3482).

**Laevissioside B:** amorphous powder; $[\alpha]_D^{26.4}$ – 62.2 (*c* = 0.18, MeOH). UV (MeOH) $\lambda_{max}$ (log $\varepsilon$): 203 (3.38), 194 (3.12) nm. IR (KBr): 3423, 2927, 1069, 1052 $cm^{-1}$. $^1H$ and $^{13}C$ NMR: see Table 2. FABMS (neg.) *m/z*: 631 $[M - H]^-$. HRESIMS (neg.) *m/z*: 667.3454 ($C_{32}H_{56}O_{12}Cl$; calcd. 667.3460).

**Laevissioside C:** amorphous powder; $[\alpha]_D^{18.9}$ – 11.2 (*c* = 0.10, MeOH). UV (MeOH) $\lambda_{max}$ (log $\varepsilon$): 201 (3.31), 191 (3.10) nm. IR (KBr): 3431, 2926, 1704, 1638, 1384, 1169, 1128, 1054 $cm^{-1}$. $^1H$ and $^{13}C$ NMR: see Table 2. FABMS (neg.) *m/z*: 777 $[M - H]^-$, 631 $[M - 146 - H]^-$. HRESIMS (neg.) *m/z*: 813.4042 ($C_{38}H_{66}O_{16}Cl$; calcd. 813.4039).

**Laevissioside D:** amorphous powder; $[\alpha]_D^{26.6}$ – 58.3 (*c* = 0.25, MeOH). UV (MeOH) $\lambda_{max}$ (log $\varepsilon$): 199 (3.07), 192 (3.12) nm. IR (KBr): 3442, 2935, 1749, 1373, 1228, 1062, 1062 $cm^{-1}$. $^1H$ and $^{13}C$ NMR: see Table 2. Acetyl groups ($\delta_C$: C=O 170.0, 170.4, 170.6, 171.0, 171.5; Me: 20.7, 20.8, 21.2, 21.2, 21.2) $\delta_H$: (1.98, 2.06, 2.13, 2.13, 2.16). FABMS (neg.) *m/z*: 987 $[M - H]^-$. HRESIMS (neg.) *m/z*: 1023.4557 ($C_{48}H_{76}O_{21}Cl$; calcd. 1023.4567).

**Acidic Hydrolysis of Compounds 1–3.** Compounds **1–3** (6–8 mg) were hydrolyzed with 2 M HCl-dioxane (1:1, 4 mL) under reflux for 6 h. The reaction mixture was extracted with $CHCl_3$ five times (4 mL × 5). The aqueous layer was neutralized with 2 M $NaHCO_3$, and was evaporated to dryness. The dry powders were dissolved in pyridine (2 mL). Then L-cysteine methyl ester hydrochloride (about 1.5 mg) was added and kept at 60 °C for 1 h. Next, trimethylsilylimidazole (about 1.5 mL) was added to the reaction mixture in ice water and kept at 60 °C for 30 min. The mixture was subjected to GC analysis, run on a Shimadzu GC-14C gas chromatograph equipped with a 30 m × 0.32 mm i.d. 30QC2/AC-5 quartz capillary column and an $H_2$ flame ionization detector with the following conditions: column temperature, 180–280 °C; programmed increase, 3 °C/min; carrier gas, $N_2$ (1 mL/min); injector and detector temperature, 250 °C; injection volume, 4 $\mu$L; and split ratio, 1/50. The configuration of D-glucose, L-rhamnose, and D-fucose were determined by comparison of the retention time of the corresponding derivatives with those of standard D-glucose, L-rhamnose, and D-fucose, giving a peak at 18.576, 16.173, and 14.865 min, respectively.

**Cytotoxic Bioassay.** The cytotoxicity assay was performed according to the MTT [3-(4,5-dimethylthiazol-2-yl)-2,5-diphenyltetrazolium bromide] method,[17] by use of the following five human cancer cell lines: Human myeloid leukemia HL-60, hepatocellular carcinoma SMMC-7721, lung cancer A-549, breast cancer SK-BR-3, and pancreatic cancer PANC-1. The $IC_{50}$ values were calculated by the Reed and Muench method.[18]

**Acknowledgments**

This work was financially supported by the National Basic Research Program of China (973 Program Nos. 2009CB522303 and 2011CB915503), the National Natural Science Foundation of China (Nos. 90813004 and U0932602), the projects from Chinese Academy of sciences (Nos. 2009311211011 and 2009312311024), and the State Key Laboratory of Phytochemistry and Plant Resources in West China (No. P2010-ZZ05).

**Open Access** This article is distributed under the terms of the Creative Commons Attribution License which permits any use, distribution, and reproduction in any medium, provided the original author(s) and source are credited.
